# How do parents access, appraise, and apply health information on early childhood allergy prevention? A focus group and interview study

**DOI:** 10.3389/fpubh.2023.1123107

**Published:** 2023-04-17

**Authors:** Jonas Lander, Eva Maria Bitzer, Julia von Sommoggy, Maja Pawellek, Hala Altawil, Cosima John, Christian Apfelbacher, Marie-Luise Dierks

**Affiliations:** ^1^Institute for Epidemiology, Social Medicine and Health Systems Research, Hannover Medical School (MHH), Hannover, Germany; ^2^Department of Public Health and Health Education, University of Education Freiburg, Freiburg, Germany; ^3^Medical Sociology, Department of Epidemiology and Preventive Medicine, University of Regensburg, Regensburg, Germany; ^4^University Children's Hospital Regensburg (KUNO), University of Regensburg, Regensburg, Germany; ^5^Institute for Social Medicine and Health Systems Research, Otto von Guericke University, Magdeburg, Germany

**Keywords:** allergy prevention, babies, infants, evidence, health information, parents, decision-making

## Abstract

**Background:**

When parents want to make health-related decisions for their child, they need to be able to handle health information from a potentially endless range of sources. Early childhood allergy prevention (ECAP) is a good example: recommendations have shifted from allergen avoidance to early introduction of allergenic foods. We investigated how parents of children under 3 years old access, appraise and apply health information about ECAP, and their respective needs and preferences.

**Methods:**

We conducted 23 focus groups and 24 interviews with 114 parents of children with varied risk for allergies. The recruitment strategy and a topic guide were co-designed with the target group and professionals from public health, education, and medicine. Data were mostly collected via video calls, recorded and then transcribed verbatim. Content analysis according to Kuckartz was performed using MAXQDA and findings are presented as a descriptive overview.

**Results:**

Parents most frequently referred to family members, friends, and other parents as sources of ECAP information, as well as healthcare professionals (HCPs), particularly pediatricians. Parents said that they exchanged experiences and practices with their peers, while relying on HCPs for guidance on decision-making. When searching for information online, they infrequently recalled the sources used and were rarely aware of providers of “good” health information. While parents often reported trying to identify the authors of information to appraise its reliability, they said they did not undertake more comprehensive information quality checks. The choice and presentation of ECAP information was frequently criticized by all parent groups; in particular, parents of at-risk children or with a manifested allergy were often dissatisfied with HCP consultations, and hence did not straightforwardly apply advice. Though many trusted their HCPs, parents often reported taking preventive measures based on their own intuition.

**Conclusion:**

One suggestion to react upon the many criticisms expressed by parents regarding who and how provides ECAP information is to integrate central ECAP recommendations into regular child care counseling by HCPs—provided that feasible ways for doing so are identified. This would assist disease prevention, as parents without specific concerns are often unaware of the ECAP dimension of issues such as nutrition.

## Introduction

Worldwide, more than one billion people suffer from allergies at least once in their life (1–5). While precise estimates for distinct allergic diseases such as asthma, hay fever, dust mite, and food allergies vary, the prevalence of allergies in infants and children is considerable, too—for example, one in eight children is affected by allergic rhinitis (6). For non-communicable, chronic diseases, there is broad consensus that research and practices focusing on prevention are key not only to reducing disease prevalence, and hence the health care burden, but also to improving the quality of life of affected individuals (7, 8). For allergies, there is evidence that preventive measures focusing on early childhood are effective, e.g., early introduction of “allergenic” foods (9).

Hence, parents have a central role in early childhood allergy prevention (ECAP), which presents at least two challenges from a research perspective. Firstly, while the shift from allergen avoidance to early exposure is well-documented and based on scientific evidence (10), various issues remain regarding what contributes to ECAP and how research findings translate into practice. These include the complexity of providing clear information to parents—for instance, that breastfeeding is strongly recommended, although it is not explicitly an allergy prevention measure (11)—and the variation among existing allergy prevention guidelines (12, 13). Secondly, previous research has found that although health care professionals (HCPs) such as pediatricians are aware of guidelines, they often fail to give respective recommendations to parents (14). Further challenges include the incomplete evidence for important aspects of ECAP, e.g., how to promote allergen tolerance in an infant's diet (15); explaining how ECAP can be practiced, e.g., how exactly to introduce allergenic nutrients in the diet (16); and the uncertainties and misunderstanding that could result from how ECAP evidence is formulated, e.g., that there is no recommendation “for or against” the use of vitamin supplements during pregnancy (17).

To overcome these challenges for effective ECAP communication and advice, we need to better understand parents' information seeking behavior regarding child health. Previous research—not specific to the field of allergy prevention—focused on what prompts parents to seek health information (18) and what the topics of information searches are (19), such as understanding the basics of asthma and how to treat symptoms (20). Moreover, research concluded that parents start their (digital) searches most frequently via Google (19) and that, while they frequently use digital sources, HCPs remain a central, trusted source of information (21). For ECAP, it seems necessary to know not only which sources parents use and why, but also how they identify them and decide which to use, how they find them helpful, and how the information influences parents' decision-making. Therefore, this study aimed to understand which sources parents access, how they appraise and apply ECAP health information, and what their respective needs and preferences are.

## Materials and methods

### Context and study design

**Figure 1:**
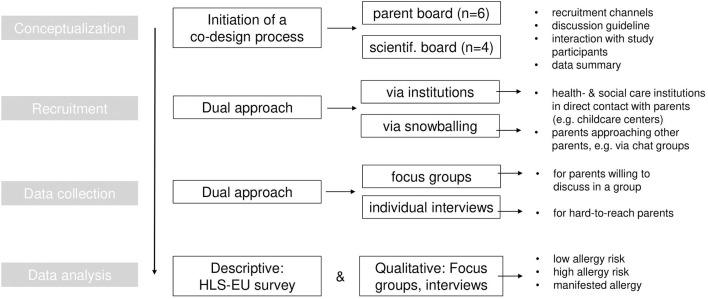
Overview of data collection and analysis.

The study is a part of the multidisciplinary and multi-center research group “Health Literacy in early childhood allergy prevention” (HELICAP, DFG FOR2959). HELICAP considers ECAP and COVID-19 in children with allergies as two cases in which health literacy (HL) is analyzed from distinct perspectives, including HCPs and target groups (parents). We explored the spectrum of perspectives, practices and needs of parents regarding ECAP via a qualitative research approach. Figure 1 gives an overview of the main methodological steps during study planning and conduct, which we reported according to the consolidated criteria for reporting qualitative research (Supplementary material 1) (22).

To plan and implement our study, we used Sorensen's health literacy model (23–25). This model puts an individual's ability to access, understand, appraise and apply health information in the context of personal and situational, as well as social and environmental determinants (26): the interplay of individual and systemic factors influences the handling of health information. We thereby developed topics for discussion and main categories to analyze the data (see below). Further, we adopted the principle of co-production (27, 28), cooperating closely with two groups to plan and implement the study: (a) we involved parents of infant children (*n* = 6), and jointly developed the recruitment strategy, discussed focus group and interview topics as well as the method of data collection, and the pre-testing of the focus groups and interviews) in four consecutive meetings; (b) we invited members of HELICAP's international scientific advisory board (*n* = 4), to integrate perspectives from public health, health education and allergology into the interview schedule. The study was conducted in accordance with the Declaration of Helsinki and approved by the Ethics Committee of Hannover Medical School (ID 8161_BO_K_2018).

### Target group

We aimed to identify expectant parents and parents of children under 3 years of age, as allergy prevention is particularly relevant in these phases of life (11). We included parents (a) with no known allergy (low risk), (b) with an allergy (meaning that child is high risk for allergy), and (c) whose child already suffers from an allergy (manifested allergic disease) to account for the spectrum of risk and disease trajectories. To explore socio-cultural and language-related differences in the search for and use of ECAP health information, we looked for parents who had arrived in Germany at most a few years ago, primarily from Arabic speaking countries and Turkey. Besides regular communication during recruitment, no prior relations existed between the researchers and the participants, who did not know any particular characteristics of the researchers besides their field of work.

### Selection of participants

Recruiting study participants is often very challenging, for instance in terms of creating awareness and interest into a specific research topic group (29–31). To address these and related challenges, we used a framework that covers central aspects of a potential participants' decision-making process, e.g., determining whether the benefits from participation outweigh the necessary efforts. We thereby aimed to focus the participants' perspective on study participation rather than researchers' considerations of what is feasible (32).

First, we compiled a list of institutions and individuals (*n* = 358) from the field of medicine (general practitioners, pediatricians, allergologists), child and social care professionals (midwives, kindergartens), and services for parents and the broader public (patient organizations, family centers) which parents of infants visit regularly. This was done for the cities of Hannover, Magdeburg, Freiburg and Regensburg, where the HELICAP research projects are located; we then extended the search to other large cities. Then, these institutions and individuals were contacted twice (in writing and electronically during April to May 2021).

As we started recruitment, the COVID-19 pandemic began and thus, we had to shift data collection as well as our recruitment channels. We intensified and formalized cooperation with a few supra-regional institutions (*n* = 5) from health and childcare. Also, we identified individuals in charge of communicating with parents within—mostly—childcare institutions near our own host institution (Hannover Medical School, Germany) and asked them to contact parents as part of their daily work. Besides this approach via institutions, we invited parents who had already agreed to participate or had already participated to spread the study call within their social networks, via e.g., WhatsApp, Facebook, or Instagram.

For all the above-mentioned recruitment channels, we provided English and Arabic translations of the study call. Specifically, we distributed leaflets in English and Arabic to those institutions identified for the recruitment within the four cities of the HELICAP host institutions. We asked these institutions to hand out the leaflet personally to potential study participants to ease the initial contact. Besides the “regular” recruitment channels, we distributed the translated leaflet within institutions and places where individuals of Arabic speaking backgrounds meet and close to our own host institution—particularly the Mosque where we approached people before and after prayer time.

### Data collection

We pre-tested a co-created semi-structured interview schedule with the members of our parents' board (*n* = 6). The schedule included a mix of regular questions and discussion topics—the same for focus groups and interviews—and more interactive case scenarios, so that participants could consider situations encountered in daily life (Supplementary material 2). The development of questions was guided by our research objectives, with input from the parent panel and the HELICAP advisory board (see above). For participants who spoke only Arabic, a native Arabic speaker created an initial written translation of the interview schedule, the study information and consent form, sought feedback on the draft as part of the pre-test and discussed potentially misleading terms within the researcher term, and then collected the data (HA).

Prior to the focus groups/interviews, each participant completed a short survey (SocSciSurvey GmbH, Germany) to collect basic socio-demographic data and allergy status (Table 1), and to assess health literacy using the 16-item short version of the HLS-EU questionnaire (Supplementary material 3) (33). Due to the COVID-19 pandemic, data was collected mostly via one-time digital focus groups (*n* = 19/23) using BigBlueButton. The host institutions' office for data safety approved the data collection. We carefully adapted data collection to the digital format, considering in particular aspects of interaction, moderation and technical prerequisites (34, 35).

**Table 1:** Socio-demographic characteristics (*n* = 114).

**Variable**		***N***	**%**
Gender	Male	6	5.3
	Female	106	93
	Other	2	1.8
Age of parents	18–29	18	15.8
	30–39	80	70.2
	40–50	15	13.2
	Mean (SD)	33.7 (7.5)	Range: 24–48
No. of children	1	61	53.5
	2	31	27.2
	3	12	10.5
	4	7	6.1
	5	1	0.9
	≥6	2	1.8
	Mean (SD)	1.8 (1.1)	Range: 1–6
Age of children (years)	< 1	52	45.6
	1– < 2	18	15.8
	2– < 3	13	11.4
	3– < 4	23	20.2
	≥4	43	37.7
Mother Language	German	72	63.2
	Arabic	16	14
	Turkish	3	2.6
	Kurdish	11	9.6
	Bilingual (German & other)	2	1.8
(School) education^*^	Low	19	16.6
	Middle	80	70.2
	High	15	13.2
Employment status	Full-time employed	20	17.5
	Part-time employed	18	15.8
	Parental leave	53	46.5
	Not employed	4	3.5
	In education (training)	10	8.8
	Permanently unable to work	1	0.9
	Housewife/homemaker	22	19.3
	Other	2	1.8
Allergy status	Low risk (no known atopic allergy)	22	19.3
	High risk (parents with atopic allergy)	51	44.7
	Manifest (child with atopic allergy)	24	21
	Family^**^	15	13
	Don't know	2	1.8

^*^Education level: low: completion of lowest possible grade (e.g., 8th grade in Germany); middle: completion of 9th/10th grade; high: all other degrees higher than 10th grade.

^**^Atopic allergy in family members, e.g., sister/brother of parent.

As various participants only agreed to an individual interview via telephone or computer, either owing to time constraints or because they preferred not to speak in a group, we provided this option (*n* = 24). When contact restrictions were lifted again and participants felt confident about personal meetings, we conducted on-site focus groups (*n* = 4). These focus groups took place in childcare institutions in more deprived city areas, to include those who do not speak German and/or those of lower socio-economic status.

Data was collected by academic researchers (MSc or PhD in Public Health), five female (HA, CJ, AM, KB, AB) and one male (JL), experienced in qualitative research methods. While no new sub-themes emerged within the main themes (Supplementary material 4) after 15 of 23 focus groups, we continued data collection to generate sufficient input from each parent group, particularly those that were more difficult to reach—as respective individuals tend to be unwilling to participate in group discussions, we offered the opportunity to participate in an individual interview instead.

### Data analysis

The pseudonymized audio files—with no additional written field notes—were analyzed according to the steps described for qualitative content analysis (36–38) using MAXQDA (Verbi GmbH, Germany). In step one, three researchers independently coded *n* = 4 transcripts (10% of the total data) for an initial understanding of the material. Arabic transcripts were translated to English (by HA) and then included for analysis.

Based on this, and according to our topic guide and research questions, we deductively derived main categories (*n* = 12), which were agreed with a fourth senior researcher, and a set of coding rules (step two). In step three, we applied the main categories to the coded transcripts. In step four, two researchers compared all coded segments according to consensual coding, meaning that any coded segment marked as unclear was discussed until consensus was reached (here: *n* = 161 text passages). Based on this, in step five we defined inductive subcategories and agreed any further addition of subcategories during the remaining coding process. We used the first full coding scheme to independently code another 20% of the material and then repeated step 3, comparing codings to achieve further agreement (discussion of *n* = 66 text passages). Step six included the coding of the remaining full material by two researchers and another exchange and comparison of any unclear text.

Overall, we structured the results according to the facets of accessing, appraising and applying health information as given in Sorensens' HL model (see above) and used this structure again to discuss how parental HL can be fostered. Given the number of participants, transcripts were not returned to them for correction after their attendance, but a summary of the findings was further discussed in a separate meeting with the parents involved in the study planning.

To allow for a descriptive summary of the ca. *n* = 2,400 coded segments, e.g., to display the frequencies and percentage shares of mentions, we assigned a numerical value to each code; for example, in the main category “ECAP information sources”, “health professional” was assigned a “1”, “Google” a “2”, “family and friends” a “3”, and so forth. This was done for each main category (*n* = 12) and split by participant group (parents with own allergy, i.e., at risk child; parents without own allergy, i.e., child not at risk; migrant parents; mixed groups) to enable comparison. We divided the coded transcripts into four further groups, to reveal potential differences among parents: (1) parents or child with atopic allergy, (2) no known allergy, (3) parents with explicit migration background, (4) mixed groups (focus groups that included parents with and without increased allergy risk for the child). For the descriptive assessment of sociodemographic and HL survey data, data sets (*n* = 114) were entered into SPSS Statistics. Regarding the HL survey data, we calculated a sum score to yield levels of HL (inadequate HL: 0–8 points, problematic HL: 9–12 points, sufficient HL: 13–16 points) based on participants' responses on a four-point scale (1 = very difficult, 2 = fairly difficult, 3 = fairly easy, 4 = very easy) (39).

### Data presentation

The qualitative findings were grouped according to four topic areas, of which the first three resemble Sorensen's HL model: (1) accessing information: sources and topics, (2) appraising information, (3) applying information (making decisions), (4) information needs and preferences; quotes for each topic are provided in separate tables.

## Results

### Participants

A total of *n* = 114 parents participated either in one of 23 focus groups (mean duration: 79 min, range: 46–124) or one of 24 individual telephone interviews (mean duration: 54 min, range: 30–66), most of them being female (*n* = 106) and often reporting a familial allergy risk or actual allergic disease (parents or child; *n* = 76). The most common reason for participants to not join the study after initial contact was lack of time. Sociodemographic details are provided in Table 1. 60.5% of participants reported difficulties in finding, appraising, or applying health information (problematic HL: *n* = 50; insufficient HL: *n* = 19). Responses to the individual HLS-EU-Q16 survey items reveal difficulties for “appraising” and “applying” health information, e.g., judging whether an information source is reliable (difficult/very difficult: 59%) or using information to make decisions (44%) (Supplementary material 5).

### Accessing information: sources and topics

Regarding ECAP, the single most cited source of information were family and friends (“peers”) (65 of 292 total mentions; 22%) (Table 2, Quote Q1), followed by “doctors” (60/292; 21%), “Google” (38/292; 13%) and social media (32/292; 11%) (Q2). Counted as a single category, HCPs were mentioned most often (86/292; 29%), and more often by migrant parents (17/51; 33%) than by the other groups (e.g., at risk group: 26/157; 16%). Parents repeatedly named pediatricians as the precise medical source, and almost never allergologists (Q3). Overall, they cited doctors much more frequently (60/292; 21%) as an information source compared to midwives (22/292; 8%).

*(…) I think the doctor is the best source of information because s/he knows your case very well. What one reads on the internet or in articles is general. (…) everyone has a particular kind of allergy, and only the doctor recognizes it and its medication. [at-risk group]*

**Table 2:** Overview of further exemplary quotes per main theme.

**#**	**Quote**	**Parent group**
	**Main theme: accessing information**	
Q1	And of course the exchange with friends, especially with people of the same age. This generation also thinks a bit differently and has a different input than, for example, grandma or mum.	Risk group
Q2	And it's so massive, Instagram and Facebook, that it's actually become a main source for me. I'm in various local groups, midwife groups, nutrition groups and follow a few psychologists on Instagram. And I'm so convinced of that, because in this little time I have as a mother, it really hits a nerve. (…) Often it's probably not that scientifically sound (…) But it sets incredibly important impulses for me (…)	risk group
Q3	(…) And I think the basic information that we have or that we also use is [what] the pediatrician says. It is simply a good relationship of trust (…) without us knowing him particularly well. But purely from his professionalism, we believe that he can assess it very well.	Risk group
Q4	Sometimes, my wife contacts her mother and sister because they have children and thus they have experience (…). So, yes, we do family discussion. And I, myself, went through some things where I called my family and asked them what to do and what things they believe could be helpful.	Migrant group
Q5	Q: Where do you search on the internet? Are there any particular websites you search for? A: On Google, of course.	Mixed group
Q6	In fact, more the subject of neurodermatitis, in terms of possible heredity [from my wife]. (…) And, yes, actually how that is inherited and a bit sensitized to the topic that we just look there, whether there is somehow something with our son.	Mixed group
Q7	So the last time, I informed myself a little bit about the nutrition regarding our daughter. Yes, what is in, so to speak, food. Also in the sense of hormones and so on, because we would like to have something checked again (…)	No risk group
	**Main theme: appraising information**	
Q8	You feel that the doctor is trusted because he knows the case he's dealing with, you know? Google tells you things in general, whether this or that.	Mixed group
Q9	This information – it's better for the person to contact his/her doctor, who gives 100% accurate information, especially that – because allergy is complex.	Migrant group
Q10	Well, I have to say that we have a really great pediatrician who always gives us lots of brochures and also really tells us a lot at every check-up (…)	Mixed group
Q11	The internet still gives you more information about the disease; I mean broader information. (…) When you Google, you get deeper and know more about the disease. So much information on the internet helps more than the doctor and his expertise. He doesn't actually tell you everything! (…) If one is free to look at a bunch of videos, s/he is going to have a whole idea of the topic and as much information about [the allergy].	Migrant group
Q12	Because with many things, especially with allergies, I have the feeling that there are always many different opinions. And sometimes it's hard for me to know who to listen to best.	No risk group
Q13	So I would think that this is so blatantly commercialised that I think it's difficult to really filter out sound information.	Risk group
Q14	But it's also something that's not explained at the pediatrician. I say you get a note for every vaccination, or what we got now, a note on how to [lay the child down correctly]. All these things, you are informed, but you are not informed about allergies for the children. Only when it's too late.	Risk group
Q15	So she didn't take it seriously, she said that children only develop allergies at a certain age, they can't have any allergies yet, that's it. And the subject was really closed for her.	Risk group
Q16	I don't actually prefer Google because I used it several times, and scary things showed up. I wasn't sleeping the whole night because of Google information [until the doctor] informed me that it's not related to my case. Since then, I didn't use Google and waited to see the results of the test. Each case has its own story. (…)	Migrant group
	**Main theme: Applying information (decision-making)**	
Q17	Oh, the midwife, she was there for us 24/7. (laughs) Such a great midwife, I really have to say, respect this job, respect this woman. She helped us so much. So you have to say, our child initially, when she was born, she had a sucking weakness (…) So regardless of that, we just always had a contact person and I just trusted her completely. And she has been in her job for a very, very long time and has a lot of experience.	Risk group
Q18	It's okay to see more than one doctor, and who has experience, can also advise you. As I said, it depends on who gives you the advice. For example, my reference is my sister–I always get back to her. She has been in Germany for a long time now, before me, and her children are older than mine. She's knowledgeable.	Migrant group
Q19	I follow up on social media, but I would never take something from them because I feel that social media is like the YouTubers who only seek more views: they come up with some ideas only to get more views. I think that what they write on social media is not accurate at all.	Migrant group
Q20	And at some point you don't want to listen to all the advice anymore and it's somehow best to just say, no, I've got this person and that person, I'll go there and that's it, right?	Risk group
Q21	Now, of course, you ask yourself again: Shit, maybe we have done exactly that/ Because allergy sufferers always say: Either you leave it out completely, and you get an allergy, or you do too much, and you get an allergy. Have we done too much?	Risk group
Q22	Allergies or allergy avoidance is an issue in that you are confronted with it when you have a small child, because then you start with complementary feeding and so on. Some say to breastfeed fully until six months. Others say to start from the fourth month and still continue breastfeeding and somehow introduce food on the side. Some say carrots, others say: please no, no carrots. (…)	No risk group
	**Main theme: accessing information**	
Q23	So, if new information or something comes up, I would want my pediatrician to tell me when I have my check-up or when I have my vaccination. Because the pediatricians are actually in the first place who have to be there for the children and have to say that, I think personally.	Mixed group
Q24	It would be important, if someone tells you at all (…) allergies in children are a topic, inform yourself about nutrition etc. (…). Just a little food for thought, that you get that already and not only when it's so far, you deal with the topic. I mean, you're informed about everything possible at the pediatrician's office (…)	Risk group
Q25	We trust [the “neighbourhood mother”], if it's not 100%, then 99.99%. Female teachers, of course, but – the one who has experience is without a doubt different.	Mixed group
Q26	(…) It was good for the parents to be able to talk about what they had experienced, because it can cause a lot of stress when the child is ill and you have to deal with the situation alone, and I think that sometimes purely factual information is not enough (…) but I think that a counterpart, whether it's the mother, a friend or the doctor, is also quite good to pick you up emotionally.	Mixed group
Q27	And nowadays (…) there are also great pediatricians who share their knowledge on the internet. And there are two that I trust very much, who also have a YouTube channel and highlights in their stories. And then, for example, I would look to see if I can find something on the topic that interests me the most right now.	No risk group
Q28	Difficult. I think it has to be worded simply, so that it is appealing to parents to know right away what it is all about. So this first website, when it's all a muddle and a lot of information, especially when you're in a situation where you know your child is not well and then you come across an information sheet like this, it simply overwhelms you. So I think the requirement would be clarity, structure, comprehensibility.	Risk group
Q29	(…) Yes, that would be a bit, I say, a bit more holistic view, wouldn't it? Not only to look at one specific topic, but also to link it with other topics, yes? Exactly, and I also believe that nutrition really is a topic that probably occupies everyone again and again, or, no, most parents do too (…)	No risk group
Q30	When I translated it to Arabic, I faced no problem: it became clearer and easier to read. Arabic is important for us, because we feel more comfortable reading it. Usually, I don't know, Google automatically translates into Arabic. So, I think that Arabic translation will exist on Google. It is better if it exists on the website: it will help us. For example, if one can't read German or his/her German is not that good, then Arabic will be so much easier to understand.	Migrant group

Parents whose child has a low risk of allergy reported relying more often on peers and less often on physicians (peers 14/45, 31%, physicians: 6/45, 13%), compared to parents who had an allergy (peers: 31/157; 20%, physicians: 26/157, 10%); migrant parents referred to peers in 12/51 mentions (23.5%) (Q4).

*For example, I also prefer to ask friends who now currently have children rather than, say, my parents' generation. [low-risk group]*

Further, Google, “the Internet” and social media together accounted for 30% of all mentions for information sources (89/292; 30%), but parents usually did not specify the precise websites or social media accounts (Q5). Also, participants from each group often did not know expert providers of digital health information specific to ECAP (not known: 40/47; 85%), e.g., the Allergieinformationsdienst (German Allergy Information Service) and the Arbeitskreis Allergiekrankes Kind e.V. (Working Group Allergy Suffering Child).

*Exactly, just type it in bluntly. What can I do to prevent my child from getting allergies, so to speak. Roughly speaking. [at-risk group]*

While we did not assess information topics explicitly, only 11% of respective mentions (5/47) related to allergy prevention. Remaining topics most often related to nutrition, allergy (not specific to prevention), and aspects of handling symptoms, e.g., atopic dermatitis (Q6, Q7). Parents already affected by allergies mentioned these topics most often (28/47; 60%), the other three groups referred to them to a lesser extent (40%).

### Appraising information

Of 183 mentions concerning parents' appraisal of information sources, 74 (40%) were positive and 109 (60%) negative. The largest share of positive evaluations (23/74; 31%) related to doctors, whose reliability and expertise parents repeatedly appreciated, along with opportunities for receiving personal feedback (Q8, Q9, Q10). Google and social media were second and third most often positively appraised (15/74; 20%, 12/74; 16%), particularly for fast, easy access, the spectrum of information topics (Q11) and, in the case of social media, “personified” and thus reliable sources.

*Yes, so kids.doc.de [paediatrician who provides child health information via Instagram], I don't think he's been doing it that long, but the reach has really exploded. It's quite good and I think it's also easy to understand for many people, so he really reaches people. [low-risk group]*

Apart from the positive appraisal of information sources, the largest share of the criticisms, i.e., negative appraisals related to Google (40/109; 37%) and social media (34/109; 31%), for which parents frequently criticized the amount of information provided, its (un)reliability and potential for creating uncertainty and groundless fear (Q12, Q13). Further, 26/109 (24%) negative evaluations related to doctors, who were criticized for not actively/explicitly addressing ECAP, providing insufficient detail, and not taking parents' concerns seriously (Q14, Q15).

*The topic of allergy is not big in Germany [and] often not taken seriously. [It's like] “oh, it's some intolerance or lifestyle”. The recommendation from the hospital was not to give hazelnuts. (…) In retrospect, that's not enough, that information and the doctors, whether you go to the pediatrician, in the hospital, or the allergist, nothing. So, you really have to get that yourself, quite actively, that's really sad, I must say. That's why I'm actually glad that a study like this is being done, so that maybe it gets a bit of a hearing, too, yes. [at-risk group]*

Looking at the different parent groups, those affected by allergies evaluated doctors negatively twice as often (15/105; 14%) as positively (7/105; 7%), with a similar frequency for Google and social media. Those with low-risk children gave most negative evaluations to Google and social media (14/40, 35%). The migrant parents' group most often positively evaluated doctors as information providers (9/46, 20%), but ascribed most of their negative evaluations to Google (10/46, 22%) (Q16), which the other groups did less often (e.g., at risk: 11%). Participants from this group perceived family and friends positively, too.

*I always ask my mother because she has experience with her children–that they went through this and that. She makes you feel less concerned or worried about your child when you don't know how to act. Mothers have plenty of information and can benefit you. [migrant group]*

To appraise the quality of information, parents used a variety of “indicators”, i.e., quality criteria. Most such mentions referred to the trustworthiness of particular information providers (“depending on the provider”) (21/92; 23%), the provider being a professional, i.e., expert (12/92; 13%), the provision of references (15/92; 16%), and providers being non-commercial (10/92; 11%). Further mentions related to information containing caveats, being neutral, up-to-date, and based on expert consensus.

Appraising information also includes the aspect of trust: Of 183 corresponding mentions, the largest portion related to trust of HCPs (84/183; 46%) (Q17), for parents of both low- and high-risk children. The migrant parents group however referred almost equally often to trust in HCPs (17/47; 36%) as well as trust in peers (14/47; 30%) (Q18). Overall, trust in peers garnered the second largest portion of mentions in this category (33/183; 18%), whereas mentions regarding distrust were most often found for digital sources, including social media (26/183; 14%). This was the case particularly within the group of migrant parents (12/47; 25%) (Q19), and least often for parents of low-risk children (3/21; 14%).

*So forums are really for amusement, but I would not look there to get info, because everyone writes everything possible. [at-risk group]*

### Applying information

While parents viewed HCPs as the most trusted source, only 18.5% of mentions (37/199; 19%) related to making a health-related decision based on what a HCP says or recommends (precisely: asking a HCP what to do in the case of contradictory information on a given topic) (Q20). While parents employed different strategies to come to a decision, including asking peers, seeking further information, comparing information, and “trial and error”, most mentions related to deciding based on intuition (79/199; 40%).

*And I think also in terms of allergy prevention, sometimes you have no choice but to follow your gut or just test it out. [at-risk group]*

This strategy was equally evident in parents of high- and low-risk children. Participants from the migrant parents group, however, most often referred to seeking an HCP's opinion to make a decision (12/40, 30%). Decision-making challenges most frequently concerned information being contradictory (20/62; 32%), not knowing which answer is right (10/62; 16%), the multitude of opinions (9/62; 15%), and the difficulty of deciding at all (8/62; 13%) (Q21, Q22). Here, parents of high-risk children expressed most of the challenges (44/64; 69%—other groups: 31%).

*Exactly, and then yes, I think there is often information, like about breastfeeding, where there are always very specific times, like six months, four months, and I think sometimes the official sources contradict each other. The WHO, for example, says something different than the federal government, for example, where some say four months of full breastfeeding, others say six. [at-risk group]*

### Information needs and preferences

When asked about who should provide information on ECAP, parents most often referred to HCPs, particularly doctors (69/100; 69%), which all parent groups favored the most. (Q23, Q24). Besides HCPs, scientific as well as social institutions, e.g., childcare and family education, were mentioned equally often (15/100; 15%) (Q25).

*We have a lot of families [in our Kindergarten] who have never used a computer, for example, and where there are language barriers, at least in German? (…) I think I also often sent links to other topics, where I always think yes, these are nicely prepared websites for the middle class. But only for the well-educated people (…) One needs to also explain it personally, because there are many sources, but the parents don't find the way. [mixed group]*

In terms of how ECAP information should be provided, parents' statements frequently related to personal, i.e., direct communication (13/54; 24%) (Q26), and digital channels such as websites (12/54; 22%) and online videos (12/54; 22%) (Q27). While those with high-risk children referred to each almost equally, migrant parents preferred personal communication and online video, which could also be understood as face-to-face interaction. Related to this, and although cultural aspects of who may communicate ECAP and how were barely mentioned, parents—particularly those with a migrant background (19/33; 57%)—repeatedly wanted information available in different languages (33/294, 11%) (Q30).

Lastly, when asked about how to communicate information, parents most often referred to structure, navigation, and clarity, i.e., the design of information materials and formats (47/118; 40%) (Q7). Besides, they emphasized the need for a basic understanding of the ways to prevent allergies in children (26/118; 22%), that information should not be prescriptive (18/118; 15%) (Q8), and that ECAP may be better integrated into other child health topics rather than communicated separately (10/118; 8%) (Q28, Q29).

*And when you read: Don't do this! Don't do that! And don't do any plants! And we have about fifty plants in the flat. Where I think they are also good for the child. But of course there's this mould risk and dust catchers and so on, it's all there. But you have doubts every day anyway (…), so it has to be formulated so that it doesn't kick you again when you're already lying on the floor, like that (…).[mixed group]*

*So maybe you could somehow integrate it into the U-Heft [parents' booklet with basic information on the child's health and examination results] or something, because people take what's in there a bit seriously. Sometimes there are also inserts for it at the pediatrician's when you have had a U-examination. [at-risk group]*

## Discussion

In this study, we explored how parents of children up to 3 years of age access, appraise and apply health information about ECAP and what their respective information needs and preferences are, by conducting focus groups and individual interviews with 114 mothers and fathers. We found that although parents frequently access digital sources, they emphasize HCPs, and also family members and friends, as important sources of information. Even though there was a lot of trust in these personal information sources, parents stated that ECAP often was not actively addressed by HCPs and that it is often difficult to distinguish good from bad advice. Parents trusted digital sources least and often did not know of evidence-based ECAP information providers. The many challenges they associate with information sources and providers often leads to their making decisions—such as which foods to give to a child at what age—based on gut feeling. The difficulties of appraising and applying ECAP information were also evident from parents' completed health literacy surveys. As parents already need to consider a range of issues related to child health, particularly when the child is still very young, many preferred to learn about it when discussing e.g., nutrition or hygiene and as part of regular consultations. We discuss specifics of these findings in the following paragraphs.

Firstly, regarding parents' ECAP information sources, the important role of peers highlights that (ECAP) disease prevention should be understood as an established social practice (40, 41). Parents obtain ECAP knowledge on baby courses, and in pediatric waiting rooms, but also from their own parents and friends, and other parents. This could be important for researchers, HCPs and health information providers, as respective professionals need to recognize (parents') knowledge acquisition and communication patterns (42). Previous research has already pointed out that HCPs need to improve their understanding of parents' health information behavior (42). Apart from parents' interactions with peers, the results clearly revealed the importance of HCPs' function as (ECAP) sources of information. As parents predominantly named pediatricians as their primary source, there could be further analysis of why midwives are much less often considered an information source, even though they are in close contact, particularly directly before and after birth. As described by von Sommoggy et al. (43), midwives often integrate ECAP only implicitly in their consultations for parents, who, in turn, may not perceive ECAP as an important subject in its own right.

Besides receiving ECAP information through personal interaction, the analysis revealed the frequent use of digital sources and social media. Particularly for Google searches and the use of “the Internet”, participants rarely specified which sources they relied on, and were largely unaware of “high quality” sources on ECAP. Given this, and since parents desired to know which specific (digital) ECAP sources to turn to, reaching a wider audience via digital sources to communicate ECAP may not work well so far. This would be important to reach parents with lower education, lower socio-economic status and/or low HL, as they could benefit most from access to reliable sources, but are the least frequent users of evidence-based health information (19, 44). For migrant parents, research showed how relevant social media is to access health information, not least to communicate in more than one language (45, 46). Here again, it would be important to explore options for how digital providers of “good” ECAP information could reach distinct audiences and target groups by offering tailored strategies (47).

Secondly, in terms of how parents evaluate ECAP information sources vis-à-vis general child health information, the most obvious difference is regarding parents' perception of HCPs, particularly doctors: while parents (very) frequently described professionals as trustworthy and appreciate face-to-face contact, the analysis repeatedly revealed criticism when it comes to ECAP. Here, parents pointed at the lack of consultation for issues that do not require immediate treatment. This is problematic as previous research found that that parents use digital sources before and after consultation with a HCP, exchange about this during consultations is missing (19), and parents' desire for more guidance on reliable information sources is not addressed (48). Parents criticize HCPs for not actively addressing, in this case, ECAP and, turn for instance to online forums as a consequence (49). As found earlier in the context of allergy guideline communication (14), HCPs often know about, but rarely communicate recommendations explicitly, hence illustrating the potential mismatch between parents' expectations and HCPs' actual (information) practice.

This problem is further highlighted by the fact that parents of children who were high-risk or had a manifested allergy often evaluated HCPs negatively, although this group is most concerned about ECAP. While previous investigations found that parents often evaluate online health information positively (50), in this study, most negative appraisals related to this type of information source. This again may be explained by the subject matter of ECAP, which is characterized by the considerable uncertainty of available evidence. If parents are confused about the quantity or quality of information, they easily turn to low-quality sources as shown by Halls et al. on the topic of eczema (49)—again, this emphasizes the importance of effective communication by the various information providers.

Thirdly, although online health information could be an important source of guidance for parents—not least given its accessibility—its current role and effect may be ambiguous. In accordance with prior findings on parental (digital) health information behavior, parents in this study reported frequently using digital ECAP sources, yet ranked digital sources least trusted of the different sources (19, 40, 51, 52). In our study, this was often justified in terms of “anyone could write anything on the internet”. Trust, however, is a crucial prerequisite for decision-making. From the perspective of parents searching for ECAP information online, awareness of quality-assured information portals specific to this subject (e.g., the German Allergieinformationsdienst) may be strengthened, not least as parents frequently expressed ignorance of such providers. A previous review by Wollmann et al. on user needs for online health information suggested that actually knowing a specific site/provider is the most crucial determinant for trusting an online source (53).

Further, as parents wished for more guidance and advice, particularly from HCPs (19, 48), future research should first investigate how HCPs could realistically raise awareness about digital information sources. It is particularly important to understand what resources and support HCPs require to act as “information mediators” or “enablers'—a role that has been previously found to strengthen individuals” HL (54)—as feasibility may be a crucial factor, considering how challenged HCPs are when taking on additional tasks. From the perspective of online health information providers and given parents' desire for navigation and orientation, official sources should better implement good practices for online health information (55, 56) to help increase trust in online sources. As parents in our study most often evaluated information in terms of who is providing it, providers need to be transparent about the provenance of information, and how a source can be identified as independent—for instance by clearly identifying sponsorship (53). This may be particularly relevant in the case of ECAP, given the changes and complexities around the “right” (i.e., evidence-based) prevention strategies.

Trust, and, more generally, how an individual accesses, appraises and applies health information should also be considered in terms of actual HL levels. Previous research shows that not only is lower health literacy associated with lower levels of trust and less use of evidence-based sources (57); HL determines both health behavior (58, 59) and knowledge about health (60). Our study did not measure if, for example, those with higher HL know more about ECAP or do more to prevent allergies in their children. However, the continuous criticisms and difficulties all parent groups expressed regarding ECAP information suggest that those with high HL are not necessarily less concerned about finding and applying correct advice. Rather, the majority of participants in our study reported difficulties with accessing, understanding, appraising and applying health information, mirroring recent representative HL statistics for the German population (61)—Improving on ECAP-related information practices therefore seems to be of widespread concern.

In terms of ECAP information needs and preferences, an important finding relates to the idea that the subject of ECAP could, in many cases, be integrated into counseling and advice, for example regarding maternal health, breastfeeding and immune system development, rather than treating it as a distinct subject. That way, parents could practice ECAP as part of their regular parenting responsibilities, without being challenged by even greater information overload—a problem highlighted several times before (62, 63). This may be particularly relevant to parents of low-risk children, who are likely to be interested in their child's health generally, but have no reason to be specifically concerned about allergies.

Parents with a familial allergy risk and migrant parents, both of whom often desired personal consultation with HCPs on the subject of ECAP, also suggested to integrate ECAP counseling into existing routine communication channels (19). The target group itself provided relevant suggestions for how to do so, for instance including ECAP information in quasi-mandatory health checks for babies and infants (German “U-examination”) and providing the information materials as part of these examinations. Outside the healthcare context, awareness of ECAP could be raised as part of child rearing and parenting education provided through childcare institutions. For hard-to-reach parents, who are particularly often excluded from effectively using online health information (60, 64), our own recruitment and the study conduct yielded important insights into how respective groups may be approached (65): in our case, “neighborhood mothers” were employed through voluntary work for childcare institutions to build trusting relationships and frequent exchange with those individuals who require regular support with, for instance, translation services and bureaucracy.

Lastly, our study revealed several aspects regarding accessing, appraising and applying (ECAP) health information that seemed to matter for culturally and linguistically diverse populations. These related for instance to the fact that an experts' advice is crucial to respective individuals (accessing information, making decisions based on information), that trust is low in Google and social media (appraising information), or that approaching them in a language they understand needs to be done better and/or more often (information needs). Such findings support the relevance of addressing the health information practices and needs of respective individuals—further research could assess how and with what detail this is done by health information providers.

## Strengths and limitations

While this was a qualitative study, its comparatively large sample size generated a comprehensive set of data. We were thus able to derive frequencies and percentages for the codings/mentions. Regarding recruitment, study participation was planned as on-site meetings at each project site, to enable lively exchange among parents on the (overall) subject of child health, often considered emotive (66). Though the COVID19-motivated transformation into a digital format could have resulted in less active debate and discussion, we took measures to compensate for this potential shortcoming, particularly the use of case scenarios to uphold interaction and exchange (67). Moreover, the considerable challenge of convincing parents facing resource and time constraints to take part in the study was eased through the shift to a digital format. Another strength of our recruitment was the targeted cooperation with child care facilities that provided specific services for “hard to reach” groups, e.g., parents from disadvantages areas. Lastly, mothers participated much more often than fathers, and therefore we asked participating mothers whether the child's father had different opinions, experiences or practices in terms of handling ECAP information. A distinct approach to generating interest and recruitment may be necessary for fathers with little interest in health information.

## Conclusion

ECAP is a useful context to assess parents' health information practices, as many reported challenges. Parents need to be sure about which advice to follow and where to find it; there are socially-rooted practices around child health and prevention, the scientific evidence is complex, and there is a wide variety of available information sources. From the perspective of Public Health, the communication of available scientific (ECAP) evidence seems crucial: health information services that, at least in some cases, already provide reliable ECAP information, may consider closer collaborations with HCPs to reach parents. Pediatricians and midwives—for instance—are in regular contact with them and could hence act as health information mediators, provided that they have feasible and effective ways for doing so. This would also be important regarding disease prevention, as parents without specific concerns are often unaware about the ECAP dimension of issues such as nutrition.

## Data availability statement

The raw data supporting the conclusions of this article will be made available by the authors, without undue reservation.

## Ethics statement

The studies involving human participants were reviewed and approved by Ethics Committee of Hannover Medical School (ID 8161_BO_K_2018). The patients/participants provided their written informed consent to participate in this study.

## Author contributions

JL and M-LD: conceptualization. JL, HA, CJ, and M-LD: data collection and analysis. JL: writing—original draft. M-LD, JS, MP, EB, and CA: writing—editing and revision. M-LD, EB, and CA: supervision and project administration. All authors have read and agreed to the published version of the manuscript. All authors contributed to the article and approved the submitted version.
