# Supplementary material for: How do parents access, appraise, and apply health information on early childhood allergy prevention? A focus group and interview study
Source: Front Public Health. 2023 Apr 17;11:1123107. doi: 10.3389/fpubh.2023.1123107 (PMC10149846; doi:10.3389/fpubh.2023.1123107)
Supplement: Supplementary file 2 [file Table_2.DOCX]

***Supplementary material***

How do parents access, appraise, and apply health information on early childhood allergy prevention? Focus group and interview study with 114 mothers and fathers

**Jonas Lander¹*, Eva Maria Bitzer², Julia von Sommoggy³, Maja Pawellek⁴, Hala Altawil¹, Cosima John¹, Christian Apfelbacher⁵, Marie-Luise Dierks¹**

***Correspondence:** Corresponding author: Jonas Lander, lander.jonas@mh-hannover.de

**Supplementary material 2, discussion guideline**

1. Welcome (5 min.)

- Welcome and thanks…
- Introduction of presenters & participants
- Background and objectives of the study…(short explanation here)
- General and organizational issues… (short summary here)

2. Topic awareness, knowledge, relevance, confidence (15 min.)

*In the introductory round, you have now already briefly reported to what extent the topic of allergies already concerns you. Once apart from that, whether there are already allergies in your family:*

(relevance, topics)

- How often do you actually deal with the topic of "allergies in children" in your everyday life?
- Is the topic rather "important" or rather "unimportant" for you? Why?
- Which topics do you deal with concretely, which questions do you have when you think about your own child?
- If allergy is not yet a major issue: are there other "child health" issues that you are more concerned with?

(Knowledge)

Have you ever read up on "allergies in children" specifically?

- If yes, what was the reason?
- What recommendations have you heard or read about when it comes to avoiding allergies?
  - Where did you get the information?
- How do you decide for yourself which advice, which recommendations you listen to or when you classify an info as "good" or "not so good"?
  - Do you think about whether information is "good" or "bad"?
- Apart from allergies, how else do you inform yourself about the topic of children's health?

Thank you very much so far! We have prepared another short example:

*Imagine you read on www.allergieinformationsdienst.de that among children and adolescents almost every fourth person suffers from an allergy.*

(Trust, Risks, Myths)

- What is your first thought when you hear this?
- What would you do to find out if it is true?
- Do you actually know the Allergy Information Service?

3. Information behavior, challenges (20 min.)

*The next part is about how you deal with health information concerning your child and how easy or difficult you find it. For this purpose, we will now post a short "case study" in the chat window, which we can also read out again....*

- Scenario A - Parents with and without special health risks for the child

Imagine you read on a well-known site on the Internet that excessive washing, cleaning, disinfecting and avoiding pets increases the risk of allergy in your child. However, you yourself are very careful about hygiene. Now you are considering whether you should change anything.

- Scenario B - Expectant and new parents

Imagine you are at the pediatrician or midwife. You talk about nutrition and learn that it doesn't make sense to avoid certain foods in babies and toddlers to reduce allergy risks. After the conversation, you want to find out more about this and read in a parenting blog that many parents avoid egg, wheat, carrots, nuts, for example (from the 5th month). Now you are not so sure what is right.

- Scenario C - Experienced parents

Imagine your child has an allergy and you have an appointment with the pediatrician. You talk about allergy treatment and learn that allergy vaccination is possible from the age of 6. At home, you want to get more information. In a forum, parents report that the therapy takes too long, is expensive and in the end doesn't help. Now you are not so sure if the doctor's info is true and if the treatment would be good for your child.

(Behavior)

- What would you do now if you wanted to look further into this info?
- Where and how would you look for more information?
- Which sources would you trust, which would you rather not?
- If you think about the Internet,
  - Which sites are helpful to you?
  - Would you also "google" this or "children and allergies" in general?
  - Do you generally also use social media such as facebook, twitter, intstagram for these topics? Why yes, no?
- What about friends, family? Do you talk about the topic?
- How do you deal with other opinions?

(Challenges)

Thinking again about weighing information (as in the example).

- What do you do when there are differing opinions?
- What do you do when there are unanswered questions?

4. Needs, future design (15 min.)

*Finally, we want to hear from you about what you think "good information" on your children's health should look like. We have prepared an example again, namely...*

(Participants read through one of the following pages (link provided by facilitator) and then discuss the following questions:

- What do you like and dislike about the page?
- Is anything missing?
- Is the info helpful to you? Why?
- If you have your own questions about children and allergies, which source would you prefer?
- Is there a particular information format that you prefer, e.g., prefer videos, newsletter, website, info directly from the doctor?
- What constitutes "good" information for you?
- Do you have examples from other areas on child health that you find good as information?
- If there were a new/different offer on the topic "How can I prevent allergies in my child?", what would you like to see? What topics and questions would you like to see there?

*(Note: this is not about this site per se, but rather more generally, what parents would like to see).*

6. Conclusion (5 min.)

*At the end, we would like to know what you "take away" from our round of talks today? Were there any topics, questions, comments that were particularly important, funny, critical, new, for you?*

7. Farewell (5 min.)

*Open questions? Reimbursement? Questionnaire online? Contacting again?*
