# Supplementary material for: How do parents access, appraise, and apply health information on early childhood allergy prevention? A focus group and interview study
Source: Front Public Health. 2023 Apr 17;11:1123107. doi: 10.3389/fpubh.2023.1123107 (PMC10149846; doi:10.3389/fpubh.2023.1123107)
Supplement: Supplementary file 3 [file Table_3.DOCX]

***Supplementary material***

How do parents access, appraise, and apply health information on early childhood allergy prevention? Focus group and interview study with 114 mothers and fathers

**Jonas Lander¹*, Eva Maria Bitzer², Julia von Sommoggy³, Maja Pawellek⁴, Hala Altawil¹, Cosima John¹, Christian Apfelbacher⁵, Marie-Luise Dierks¹**

***Correspondence:** Corresponding author: Jonas Lander, lander.jonas@mh-hannover.de

**Supplementary material 3, participant survey**

Short questionnaire for the study "How do parents inform themselves about children's health? In the first part, we would like to ask you some questions about how you deal with health information.

| We are interested in how easy or difficult you find it to find, use and evaluate health information. Please mark the answer that applies most to you. | | | | | |
| --- | --- | --- | --- | --- | --- |
| On a scale from very easy to very difficult, how easy / difficult do you think it is... | Very difficult | Rather difficult | Rather easy | Very easy | Don’t know |
| find information on treatments of illnesses that concern you? |  |  |  |  |  |
| find out where to get professional help when you are ill? |  |  |  |  |  |
| understand what your doctor says to you? |  |  |  |  |  |
| understand your doctor’s or pharmacist’s instruction on how to take a prescribed medicine? |  |  |  |  |  |
| judge when you may need to get a second opinion from another doctor? |  |  |  |  |  |
| use information the doctor gives you to make decisions about your illness? |  |  |  |  |  |
| follow instructions from your doctor or pharmacist? |  |  |  |  |  |
| find information on how to manage mental health problems like stress or depression. |  |  |  |  |  |
| understand health warnings about behaviour such as smoking, low physical activity, drinking too much? |  |  |  |  |  |
| understand why you need health screenings? |  |  |  |  |  |
| judge if the information on health risks in the media is reliable? |  |  |  |  |  |
| decide how you can protect yourself from illness based on information in the media? |  |  |  |  |  |
| find out about activities that are good for your mental well-being? |  |  |  |  |  |
| understand advice on health from family members or friends? |  |  |  |  |  |
| understand information in the media on how to get healthier? |  |  |  |  |  |
| judge which everyday behaviour is related to your health? |  |  |  |  |  |

Finally, please give us some information about yourself.

| 1. | How many children live in your household? 🖋 _________ children | | | |
| --- | --- | --- | --- | --- |
| 2. | How old are your childre? 🖋 _________ years old | | | |
| 3. | Are there any allergies in your family?  (myself, my partner, my child, someone else in my family) | | | |
|  | *Yes, namely: 🖋* | | | |
|  | *No* | | | |
|  | *Don’t know* | | | |
| 4. | Do you, your partner or your child have another chronic disease?  (for example: COPD, cardiovascular disease, diabetes) | | | |
|  | *yes* | | | |
|  | *no* | | | |
|  | *Don’t know* | | | |
| 5. | What is your gender? | male female diverse | | |
| 6. | How old are you? 🖋 _________ years old |  | | |
| 7. | What is your mother language? ___________ 🖋 | | | |
| 8. | What is your highest school-leaving qualification or university degree? | | | |
|  | (still) without a school-leaving qualification | | | |
|  | *Elementary/secondary school leaving certificate or polytechnic secondary school leaving certificate 8th or 9th grade* | | | |
|  | *Secondary school leaving certificate, Realschulabschluss or Polytechnische Oberschule with 10th grade certificate* | | | |
|  | *Advanced technical college entrance qualification (completion of a technical college, etc.)* | | | |
|  | *Abitur or Erweiterte Oberschule with completion of grade 12* | | | |
|  | *University of applied sciences degree / university degree* | | | |
|  | *A different one, namely: __________🖋* | | | |
| 9. | How is your working life or your domestic and family work at the moment?  Please tick all the answer options that apply to you. | | | |
|  | *Employed (full-time)* | | ** | *Emloyed (part-time)* |
|  | *Various employments (part-time)* | | ** | *unemployed* |
|  | *In training (school, university)* | | ** | *Permanently on sick leave / unable to work* |
|  | *Pensioner* | | ** | *Housewife / Househusband* |
|  | *A different one, namely: 🖋* | | | |
