# Supplementary material for: How do parents access, appraise, and apply health information on early childhood allergy prevention? A focus group and interview study
Source: Front Public Health. 2023 Apr 17;11:1123107. doi: 10.3389/fpubh.2023.1123107 (PMC10149846; doi:10.3389/fpubh.2023.1123107)
Supplement: Supplementary file 4 [file Table_4.DOCX]

***Supplementary material***

How do parents access, appraise, and apply health information on early childhood allergy prevention? Focus group and interview study with 114 mothers and fathers

**Jonas Lander¹*, Eva Maria Bitzer², Julia von Sommoggy³, Maja Pawellek⁴, Hala Altawil¹, Cosima John¹, Christian Apfelbacher⁵, Marie-Luise Dierks¹**

***Correspondence:** Corresponding author: Jonas Lander, lander.jonas@mh-hannover.de

**Supplementary material 4, Main themes and sub-themes**

| **Main theme** | **Subtheme level 1** | **Subtheme level 2** |
| --- | --- | --- |
| 1) Early childhood allergy prevention (ECAP) relevance |  |  |
|  | Relevant | - |
|  | Relevant if necessary | - |
|  | Little, not relevant | - |
|  | Child health more generally relevant | - |
|  | Other | - |
| 2) ECAP practices |  |  |
|  | Curing symptoms | - |
|  | Preventive action | - |
|  | Only when necessary | - |
|  | General child health practices | - |
|  | No specific actions | - |
| 3) ECAP knowledge |  |  |
|  | Which knowledge? | Prevention strategies |
|  |  | Allergy causes |
|  |  | Child health |
|  |  | Other |
|  |  | No knowledge |
|  | Knowledge sources? | Expert knowledge |
|  |  | Own experience |
|  |  | Heard or read about it somewhere |
|  |  | other |
| 4) ECAP info access |  |  |
|  | Topics | - |
|  | Sources | HCPs |
|  |  | Peers |
|  |  | Google |
|  |  | Social media |
|  |  | Midwifes |
|  |  | “Internet” |
|  |  | Non-digital media |
|  |  | Websites |
|  |  | Other HCP |
|  |  | Other |
|  | Info case study | Would consult HCP |
|  |  | Would consult digital sources |
|  |  | Would consult peers |
|  |  | other |
| 5) ECAP info appraisal |  |  |
|  | positive | HCP |
|  |  | Social media |
|  |  | Google, Internet |
|  |  | Peers |
|  |  | midwifes |
|  |  | other |
|  | negative | HCP |
|  |  | Social media |
|  |  | Google, Internet |
|  |  | Peers |
|  |  | midwifes |
|  |  | other |
|  | Website known | - |
|  | Website unknown | - |
|  | What is a “good” source? | Depends on who provides it |
|  |  | Public, official provider |
|  |  | Scientific provider |
|  |  | Provides sources |
|  |  | Self-critical |
|  |  | Consensus-based |
|  |  | Non-commercial |
|  |  | Up-to-date |
|  |  | neutral |
|  |  | Imprint info |
|  |  | other |
| 6) ECAP info application (decision-making) |  |  |
|  | How | By myself |
|  |  | By expert opinion |
|  |  | By asking family, friends |
|  |  | By trying out |
|  |  | By comparing |
|  |  | By searching further |
|  |  | By additional opinion |
|  |  | By myself |
|  | Challenges | Conflicting statements |
|  |  | Too many statements |
|  |  | Knowing what is true |
|  |  | Making a decision |
|  |  | emotionality |
|  |  | comprehension |
|  |  | Other |
| 7) Comparison with partner  (mother, father) | My partner handles ECAP/child health information differently |  |
|  | We handle ECAP/child health jointly |  |
|  | Other |  |
