# Supplementary material for: How do parents access, appraise, and apply health information on early childhood allergy prevention? A focus group and interview study
Source: Front Public Health. 2023 Apr 17;11:1123107. doi: 10.3389/fpubh.2023.1123107 (PMC10149846; doi:10.3389/fpubh.2023.1123107)
Supplement: Supplementary file 5 [file Table_5.DOCX]

***Supplementary material***

How do parents access, appraise, and apply health information on early childhood allergy prevention? Focus group and interview study with 114 mothers and fathers

**Jonas Lander¹*, Eva Maria Bitzer², Julia von Sommoggy³, Maja Pawellek⁴, Hala Altawil¹, Cosima John¹, Christian Apfelbacher⁵, Marie-Luise Dierks¹**

***Correspondence:** Corresponding author: Jonas Lander, lander.jonas@mh-hannover.de

**Supplementary material 5, health literacy scores**

Distribution of HLS-EU-Q16 overall scores according to education

| ***Variable/ Thresholds of HL levels** | **Total (n=114)** | **High education**  **(n=88)** | **middle education**  **(n=15)** | **Low education**  **(n=10)** |
| --- | --- | --- | --- | --- |
| General HL |  | MW=11.72  Median=12  (2,54) | MW=10.46  Median=10  (3,13) | MW= 10.7  Median=11  (3,05) |
| Inadequate  (0-8) | 19 | 11 | 5 | 3 |
| Problematic  (9-12) | 50 | 41 | 5 | 3 |
| Sufficient  (13-16) | 45 | 36 | 5 | 4 |

*HLS-EU-Q-16 Score-thresholds: 13–16 = sufficient, 9–12 = problematic, 1–8 = inadequate.

Results of the health literacy survey (HLS-EU-Q16)

| **On a scale from very easy to very difficult. how easy would you say it is to:** | **easy (1)** | **difficult (0)** | **Don’t know** |
| --- | --- | --- | --- |
| **Find/obtain/ access information** | | |  |
| 1. find information on treatments of illnesses that concern you? | 83 | 28 | 3 |
| 2. find out where to get professional help when you are ill? | 95 | 18 | 1 |
| 8. find information on how to manage mental health problems like stress or depression. | 36 | 58 | 20 |
| 13. find out about activities that are good for your mental well-being? | 92 | 16 | 6 |
| **Understand Information** |  |  |  |
| 3. understand what your doctor says to you? | 91 | 23 |  |
| 4. understand your doctor’s or pharmacist’s instruction on how to take a prescribed medicine? | 105 | 8 | 1 |
| 9. understand health warnings about behaviour such as smoking, low physical activity, drinking too much? | 102 | 6 | 6 |
| 10. understand why you need health screenings? | 98 | 7 | 9 |
| 14. understand advice on health from family members or friends? | 96 | 13 | 5 |
| 15. understand information in the media on how to get healthier? | 84 | 25 | 5 |
| **Appraise /evaluate information** |  |  |  |
| 5. judge when you may need to get a second opinion from another doctor? | 52 | 59 | 3 |
| 11. judge if the information on health risks in the media is reliable? | 50 | 59 | 5 |
| 16. judge which everyday behaviour is related to your health | 92 | 18 | 4 |
| **Apply/ use information** |  |  |  |
| 6. use information the doctor gives you to make decisions about your illness? | 69 | 44 | 1 |
| 7. follow instructions from your doctor or pharmacist? | 105 | 7 | 2 |
| 12. decide how you can protect yourself from illness based on information in the media | 53 | 57 | 4 |
